# Syllabus of a Course of Lectures on Surgery

**Published:** 1834-01-01

**Authors:** 


*Syllabus of a Course of Lectures on the Principles and Practice of Surgery*. By FREDERICK TYRRELL, Esq., Surgeon to St. Thomas's Hospital, and to the London Ophthalmic Infirmary. —London, 1833. 8vo. pp. 116.

THIS is a very good syllabus, clear, complete, and well-arranged; and, though a syllabus can only be considered as a skeleton, yet, as in other skeletons, its symmetry is a witness of the perfection of the body to which it belongs. A simile is not expected to be complete in all its parts, and we trust therefore that it may be long before Mr. Tyrrell's lectures are defunct. Many persons who have not the advantage of profiting by the author's oral instructions, might, we think, advantageously use this abstract of them, as a guide to their surgical studies.

